# Bright, noniridescent structural coloration from clay mineral nanosheet suspensions

**DOI:** 10.1126/sciadv.abl8147

**Published:** 2022-01-26

**Authors:** Paulo H. Michels-Brito, Volodymyr Dudko, Daniel Wagner, Paul Markus, Georg Papastavrou, Leander Michels, Josef Breu, Jon Otto Fossum

**Affiliations:** 1 Department of Physics, Norwegian University of Science and Technology (NTNU), Trondheim, Norway.; 2 Department of Inorganic Chemistry I and Bavarian Polymer Institute, University of Bayreuth, Bayreuth, Germany.; 3 Department of Physical Chemistry II and Bavarian Polymer Institute, University of Bayreuth, Bayreuth, Germany.

## Abstract

Structural colors originate by constructive interference following reflection and scattering of light from nanostructures with periodicity comparable to visible light wavelengths. Bright and noniridescent structural colorations are highly desirable. Here, we demonstrate that bright noniridescence structural coloration can be easily and rapidly achieved from suspended two-dimensional nanosheets of a clay mineral. We show that brightness is enormously improved by using double clay nanosheets, thus optimizing the clay refractive index that otherwise hampers structural coloration from such systems. Intralayer distances, and thus the structural colors, can be precisely and reproducibly controlled by clay concentration and ionic strength independently, and noniridescence is readily and effortlessly obtained in this system. Embedding such clay-designed nanosheets in recyclable solid matrices could provide tunable vivid coloration and mechanical strength and stability at the same time, thus opening a previously unknown venue for sustainable structural coloration.

## Teaser

Nematics of clay mineral double nanosheets provide very bright, noniridescent structural colors in a transparent matrix.

## INTRODUCTION

Structural colors arise when photonic waves interfere constructively following reflection and scattering from nanostructures with distances comparable to wavelengths of visible light (*1*). The structural coloration mechanism is fundamentally different from the absorbance of dyes or pigments. With structural colors, the material might be semitransparent, and the color spectrum may be tuned by adjusting the nanostructures. This mechanism, most often in combination with light-absorbing dark pigments, is a major biological coloration mechanism found in nature, featured in birds, marine animals, some mammalian species, insects, and certain plants (*2*–*8*).

Bright and noniridescent structural colorations are highly desirable. Several approaches to obtain them are reported in the literature. Gabriel *et al.* (*9*) used a lamellar system of solid acids to obtain a nematic phase that can be aligned using a magnetic field and displays a bluish color. More recently, Mouri *et al.* (*10*), working on niobate nanosheets, showed structural colors by adjusting the electrolyte concentration. Structural colors can also be obtained using block copolymer micelles, which can have tunable particle sizes and be dried and stored (*11*). Bioinspired cellulose-based materials have also demonstrated this capacity (*11*, *12*). In addition, as discussed by Barty-King *et al.* (*13*), small amounts of biodegradable replacements, such as polystyrene particles, are not necessarily harmful, as long as they are fully functional replacements.

Structural coloration sparks an enormous effort within the industrial sector to incorporate them into everyday products. L’Oreal’s photonic cosmetics and Morphotex are representative bioinspired designs (*14*). The Lexus LC blue (*15*) is a spectacular example of scaled-up structural coloration; however, according to Lexus, it takes 8 months to fabricate a sufficient quantity of pigments to cover 300 cars (*15*). The pigment’s fabrication time and abundance are major and general obstacles for industrial upscaling. Fast and simple preparation together with incorporation of abundant sustainable materials in industrial structural coloration could promote upscaling and help to achieve the goals of circular economy.

One factor of importance for structural coloration is iridescence (*3*, *5*–*7*). It is related to the quality of periodicity realized in the one-dimensional (1D), 2D, or 3D photonic crystals (*1*, *7*, *16*, *17*). For the noniridescent colors, some degree of disorder should be present at the nanoscale level (*16*). One example of this is the feathers of blue birds and the wings of blue butterflies, which were successfully mimicked using colloidal particles (*4*).

As an alternative to high refractive index monodispersed solid pigments, soft materials also show structural coloration. Liquid crystalline phases are intermediate between well-ordered crystalline solids and disordered liquids or glassy structures and thus can provide photonic structures that can be iridescent or noniridescent. Chiral structures of nanocellulose rod systems (*18*, *19*) and 1D photonic Bragg stacks, including those obtained from nematic or smectic assemblies of colloidal platelets (*10*, *20*–*24*), are well-established liquid crystalline examples of structural colors. Contrary to colors from the opal-like structures, for which a change in the size of the building blocks is required to tune colors, in liquid crystals, this just needs changing the separation between the scattering particles (*25*–*28*).

Unfortunately, structural colors could not yet be realized for the most abundant 2D material, clay minerals. Clay minerals are sustainable in terms of natural abundance and stability over geological time scales (*29*), their nontoxic properties (*30*), and consequently their wide range of established and developing applications (*31*, *32*), which render them most appropriate for upscaling. Studies on the toxicity of clay minerals have been performed. They show that there is no systemic toxic effect when digested (*30*, *33*), while airborne clay could be harmful to the lungs (*33*).

**Fig. 1.**
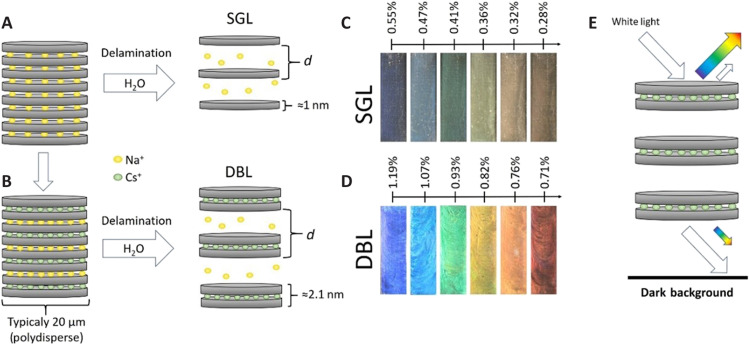
Principle of production of structural colors from nematic clay double layers (DBLs). (**A**) Schematic of the 2D lamellar structure of synthetic Na-fluorohectorite (Na-FHt). Na-FHt spontaneously forms nematic phases of single 1-nm-thick nanosheets [single layers (SGLs)] when immersed into water. (**B**) Schematics of protocol for production of nematic phases of double 2-nm-thick layers (DBLs). (**C**) Structural colors obtained from SGL aqueous suspensions at zero ionic strength. (**D**) Structural colors from DBL aqueous suspensions at zero ionic strength. The clay concentrations are given in volume %. (**E**) Principle of reflective structural coloration obtained from a lamellar Bragg stack suspension. Each lamella is semitransparent, reflecting part of the incoming white light that then interferes constructively according to Bragg-Snell’s law, thus enhancing a single color that is both dependent on the layer distance and the angle of observation (iridescence). A dark background absorbs the white light that is transmitted through the whole stack. Only the DBL case is shown in the sketch.

The most promising subclass of clay minerals in this context are swelling 2:1 layered silicates (fig. S1) (*34*), yielding solid nanosheets with 1 nm thickness (fig. S2A). These nanosheets carry a net negative charge compensated by cations associated with the surfaces (Fig. 1). Depending on the type of surface cation, the charge density, and the suspension medium, they may show the phenomenon of repulsive osmotic swelling, by which nematic liquid crystalline phases are formed (*35*–*40*).

Na-fluorohectorite (Na-FHt) is a synthetic clay mineral with superior quality regarding structural homogeneity, narrow charge distribution, and especially large aspect ratio (≈20,000) compared to most other clay minerals (*41*–*43*). Na-FHt when immersed in water forms nematic suspensions where single nanosheets are separated to uniform distances defined by the amount of water added (*39*, *43*–*46*), as evidenced by small-angle x-ray scattering (SAXS), birefringence (BF), or other methods (*36*, *37*, *39*, *45*, *47*–*50*).

## RESULTS

Here, we demonstrate that by simply tuning the Na-FHt/water ratio, nanosheet separations corresponding to the wavelength range of visible light, photonic Bragg stacks covering the whole spectrum of rainbow colors can be produced easily and rapidly. Suspended single layers (SGLs) give smooth colors of mediocre brightness (Fig. 1C). However, the brightness and noniridescence of the structural colors of the clay photonic structures can be improved enormously by applying double layers (DBLs) of two SGLs pinned together by Cs^+^ (Fig. 1D and fig. S2 B). The DBLs were obtained following a previously developed protocol for producing ordered interstratifications of Na^+^ and Cs^+^ (fig. S3). When immersed into water, nematic phases of DBLs are obtained within seconds to a few minutes by repulsive osmotic swelling (Fig. 1B) (*46*, *51*), thus producing structural coloration rapidly. There might be a direct biomimetic analog to this mechanism: Loliginid squids are able to reversibly tune their structural colors by the osmotically driven changes to the Bragg lamellar stacks in the iridocyte cells (*52*).

Furthermore, ionic strength provides an additional parameter that controls the photonic response. Structural coloration from the DBLs relies on strong electrostatic repulsion between cofacial clay nanosheets of huge aspect ratio in nematic aqueous suspensions, allowing to separate them to various distances simply by adding the right amount of water, in this way choosing the wavelength that is going to interfere constructively. The constructive interference of white light from individual nanosheets is described by the Bragg-Snell’s law (fig. S4), 2*d*(*n*^2^ − sin^2^θ)^1/2^ = *m*λ (*23*, *24*, *28*, *53*), where *d* is the nanosheet separation and θ is the angle between the observer’s point of view and the nanosheet plane. *n* = (*n*_1_^2^Φ_1_ + *n*_2_^2^Φ_2_)^1/2^ is an effective refractive index (*28*), where *n*_1_ and *n*_2_ are refractive indexes and Φ_1_ and Φ_2_ are volume fractions of each component (water and FHt). *m* is the order of the Bragg-Snell reflection and λ is the wavelength of the light enhanced by constructive interference. The color observed depends on the layer distance and, in general, on the angle of observation (iridescence). A black substrate must absorb the white light that is transmitted through the whole Bragg stack to prevent the reflection of white light that will fade the brightness of the structurally enhanced color.

Following the principle displayed in Fig. 1, we control nanosheet separation by tuning the clay concentration in suspensions in flat quartz cuvettes with a 1-mm path length. Movie S3 shows the rapid tunability of the structural colors by adding water in the suspension.

**Fig. 2.**
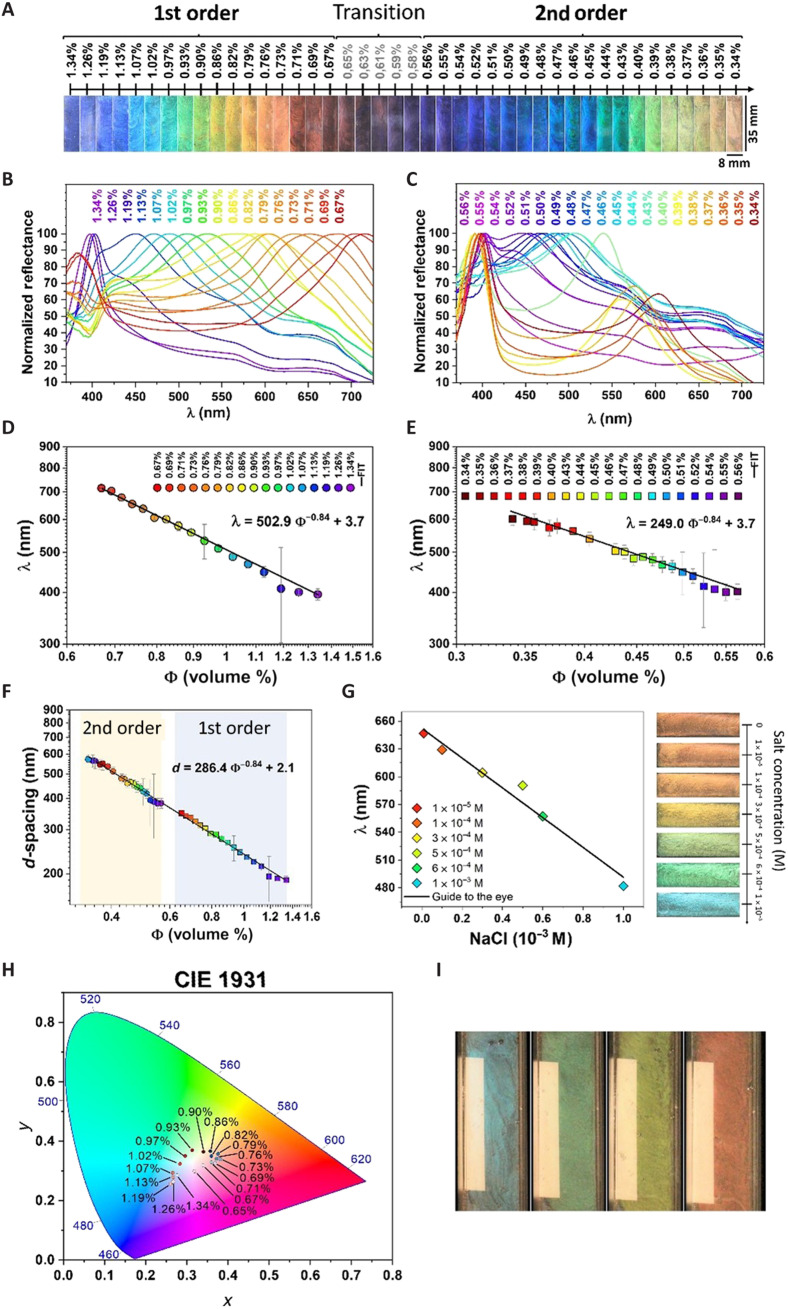
Characterization and control of structural colors from nematic clay DBLs. (**A**) Structural colors of the R1 and R2 ranges (fig. S6 shows the birefringence). (**B**) RSP for R1 range. (**C**) RSP for R2 range. (**D**) RSP maxima (with error bars) versus volume % and the linear fit. (**E**) RSP maxima (with error bars) versus volume % and the linear fit. Details of how the RSP maxima were determined and how the errors were estimated from these fits are explained in fig. S7. (**F**) *d*-Spacing (with error bars) versus volume % obtained from R1 and R2 ranges and linear fit. (**G**) RSP maxima versus ionic strength and corresponding observed structural colors. (**H**) CIE (Commission Internationale de l’Elcairage) diagram of the first-order colors. (**I**) Effect of dark and white backgrounds, respectively; see also movies S1 and S2.

DBL suspensions present two separate structural color ranges corresponding to *m* = 1 and *m* = 2, respectively, in the Bragg-Snell equation (Fig. 2A), the first range (R1) from 1.34 to 0.67 volume % (Fig. 2, B and D) and the second range (R2) from 0.56 to 0.34 volume % (Fig. 2, C and E). Using Bragg-Snell’s law for normal incidence/observation *d* = ½ *m*λ(*n*^2^ – 1)^1/2^, the *d*-spacings are calculated for first- and second-order colors. In principle, the effective refractive index could be determined using SAXS and reflectivity spectrophotometer (RSP) data in the Bragg-Snell’s law, but the broad SAXS peaks (fig. S5) with corresponding large uncertainties in the estimate of *d* makes this impossible. Moreover, because all DBL suspensions consist primarily of water (>98%), the best estimate is that the effective *n* ≈ *n*_water_ = 1.33 (*28*).

**Fig. 3.**
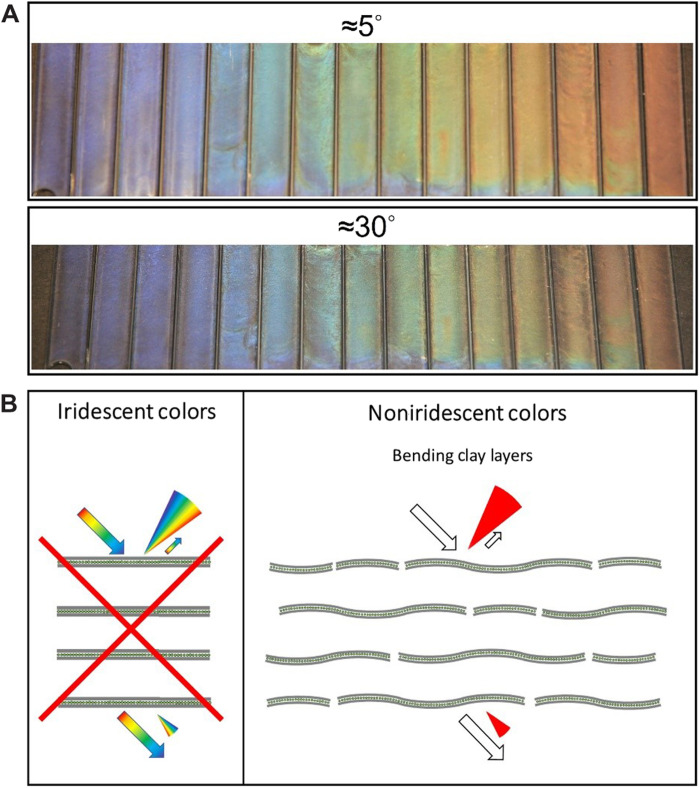
Noniridescent structural colors from nematic clay DBLs. (**A**) Structural colors at different angles (5° and 30°). (**B**) Sketch of structural order that would give iridescent colors and sketch of possible factors of disorder that, in combination, may explain the observed noniridescence color.

Using *n* ≈ 1.33, the *d*-spacings obtained from the spectral data (Fig. 2F) can be calculated for all the concentrations. The fits indicate the existence of a power law, Φ^−0.84^ behavior covering both the R1 and R2 range. This type of power law was previously observed (*32*) and was suggested to be due to the expected increased disorder when the clay suspension is diluted, such as the sketch in Fig. 3B.

Because electrostatic interactions govern nanosheet separation, the colors can also be tuned by varying the ionic strength. When gradually increasing the ionic strength of a red DBL suspension (0.71 volume %), the structural color is blue-shifted as the nanosheet separation decreases because of increasing electrostatic screening (Fig. 2G). A nearly linear relation between the RSP wavelength peak and the salt concentration is observed. Linear dependence indicates that these *d*-spacings are not determined solely by the Debye screening length (κ^−1^), for which a square root dependence on the monovalent ionic concentration is expected. This could possibly be related to the Φ^−0.84^ behavior described above. Figure 2H displaces the CIE (Commission Internationale de l’Elcairage) diagram for the first-order colors. Figure 2I shows that a light-absorbing dark background is necessary to observe the colors.

Quite unexpectedly, all our samples appear noniridescent to the eye. This is illustrated by pictures taken at two different angles (5° and 30°) (Fig. 3A). Only by close inspection is it possible to notice slight differences in the brightness of the colors depending on the angle of view. We suggest that, for the nematic clay nanosheet suspensions, the structural colors are nearly noniridescent because of a combination of local disorders related to nanosheet bending and wrinkling (*45*) and the turbostratic organization in the plane of nanosheets. In other colloidal systems, it has been demonstrated that noniridescence can originate from factors such as rapid assembly, binary colloidal mixing, or by using polydisperse systems with less than 10% polydispersity (Fig. 3B) (*54*). Clay nanosheets are strong in-plane but rather soft cross-plane, allowing them to curve or undulate (*46*, *51*). Adjacent layers of clay nanosheets in the Bragg stacks that can be turbostratically stacked with random displacement (*43*). This will result in local variations of separations, while the moment of inertia of the clay layers is evenly spaced.

The samples were studied in fixed spaced quartz cuvettes. Note that sealed samples resting “on the desk” for more than 4 to 5 days began to exhibit some iridescence and that, for samples prepared in saline solution, this degradation time was shorter, in the order of 2 days (fig. S8). This is due to sedimentation of the suspensions, which modify the colors. The colors are rapidly recovered after gentle shaking of the cuvettes. However, these time scales are more than sufficient for fixing the noniridescent nature of structural colors in a transparent matrix and subsequent industrial roll to roll processing before pigment fabrication (*47*, *48*). Another important factor in the context of applications is the reduction of thickness (*4*) below 1-mm films. In fig. S9, we show colors obtained in 200-μm-thick suspensions.

We note that a crucial factor for the brightness of these structural colors is the use of a black light-absorbing background beneath the Bragg-Snell stacks because these structural colors are practically semitransparent and, in the transmission point of view, exhibit a bright white light. Thus, it would be important for certain applications to replace the light-absorbing background with absorbing particles embedded directly in the Bragg-Snell stack. This has been done previously with success for structural coloration from systems of packed spherical colloidal particles (*55*).

## DISCUSSION

Because of the sustainability and abundance of clay minerals, the present system carries considerable potential for upscaled applications in various areas ranging from pigments in cosmetics and health applications to windows and tiles. The results and understanding obtained here on synthetic clays should be transferred to natural clays, where vermiculite (*31*, *32*), due to its large aspect ratio, presents itself as the most suitable candidate for upscaling the concept presented here. It is well known that exfoliated clay nanosheets, when added in small amounts to polymer matrices (such as degradable biopolymers) or to hydrogel matrices, can enhance considerably and can be used to tune mechanical strength and stability of the resulting composites (*56*–*70*). Thus, our results could break new ground when embedding appropriate amounts of these clay nanolayers into transparent but otherwise mechanically weak matrices, providing structural coloration, mechanical strength, and tunable stability at the same time. Our results could have a particular impact in areas such as for cosmetics and personal care applications, where new regulations currently force the industry to make use of more sustainable and recyclable formulations. In this way, these results have the potential of promoting sustainable upscaling and help to achieve the goals of circular economy.

In future work, we will exploit different matrices, such as biobased polymers to achieve sustainable and biodegradable pigments. In addition, by using responsive polymers or dopants, we may be able to fabricate externally switchable colors (electric, magnetic, and mechanical). As the clay DBLs are only a minor ingredient in these photonic structures and the vast majority of the volume is taken by the fixation matrix, the final pigment price will be specified by the price of the optimal matrix.

## MATERIALS AND METHODS

### Materials

[Na_0.5_]^inter^[Mg_2.5_Li_0.5_]^oct^[Si_4_]^tet^O_10_F_2_ was obtained by melt synthesis followed by long-term annealing according to a published procedure (*41*). The material featured a cation exchange capacity of 1.27 mmol/g and a density of 2.73 g/cm^3^. CsCl (ReagentPlus, ≥99.9%), NaCl (EMSURE ACS, ISO, Reag. Ph. Eur, ≥99.5%).

### Structural color suspensions

The clay concentrations are expressed as volume fraction in units of percentage (Φ). Na-FHt SGL stock suspensions (SGS) at Φ = 0.72% were produced by spontaneous exfoliation in water governed by repulsive osmotic swelling of sodium interlayers.

The Cs-FHt DBLs were obtained by ordered interstratification following the protocol described in (*35*), which constitutes a partial ion exchange of interlayer sodium cation with cesium, resulting in an ordered interstratified heterostructure. When dispersed in water, the sodium interlayer undergoes repulsive osmotic swelling, resulting in DBL suspensions. DBL stock suspension (DS) at Φ = 1.34% was produced by centrifugation. Subsequently, solutions of varying concentrations (1.26 to 0.56%) were prepared using 400 μl of DS and addition of deionized water (25 to 1100 μl). The SGL suspension was prepared in varying concentrations (0.64 to 0.25%) using 400 μl of SGS and the addition of deionized water (25 to 700 μl). Then, the samples were put in an IKA overhead shaker at 50 rpm for 10 min. Following this, the various suspensions were inserted by means of a syringe in Hellma quartz cuvettes with 1-mm path length suitable for RSP and BF measurements.

### Influence of ionic strength

The structural color suspensions were prepared using 400 μl of DS and 350 μl of NaCl saline solutions (from 1 × 10^−5^ to 3 × 10^−3^ M), yielding a suspension at 0.71 volume %, and in addition, a control sample was prepared using deionized water. Then, the samples were put in an IKA overhead shaker at 50 rpm for 10 min and, after that, inserted by means of a syringe in Hellma quartz cuvettes with 1-mm path length suitable for RSP measurements.

### Small-angle x-ray scattering

The samples were prepared from DS by increasing the concentration by centrifugation at 14,000 rpm to obtain a viscous gel. Samples with concentrations of 1.34, 2.56, 4.29, 5.56, and 7.21%, respectively, were filled in 1-mm glass capillaries (Hilgenberg, code 4007610). SAXS data were collected at the Norwegian University of Science and Technology using an in-house x-ray scattering instrument equipped with a Xenocs x-ray microsource with a copper anode (energy of 8 keV, λ = 1.54056 Å) and a PILATUS3 200K (DECTRIS) detector positioned at two different sample-detector distances, approximately 1 or 0.2 m, respectively. The instrument was calibrated with silver behenate. Background scattering contributions coming from capillary walls, water, and instrument atmosphere [helium (1 m) or air (0.2 m)] were subtracted from each measurement. The data were analyzed using SasView version 5.0.4 (*71*).

### Reflectivity spectrophotometer

Data were collected using a commercial integrating sphere spectrometer Avantes model AvaSpec-ULS2048Cl-EVO, with an available wavelength range of 200 to 1100 nm. The white light source used was Avantes AvaSphere-50-LS-HAL-12V, with a wavelength range of 360 to 2500 nm and a color temperature of 2850 K. The samples in quartz cuvettes were placed horizontally underneath the integrating sphere on top of a black light-absorbing background. The same black background was used for all the samples. Measurements were made at normal incidence/reflection (sinθ = 1 in Bragg-Snell’s law). First, we measured the RSP of deionized water with standard white and black backgrounds to calibrate the spectrometer. The clay suspensions produced bright structural colors that were measured immediately after filling the cuvette. The reflection spectrum of each sample has a characteristic wavelength peak.

### Photos

After RSP measurements, the samples were placed horizontally on top of the same type of black background, and photos were taken using a Canon EOS 550D camera with objective lens Sigma DC 17–70 mm and a Zeiss KL 150 LCD light source, color temperature 3000 K.

### Birefringence

Data were collected by placing the same cuvette samples used for the RSP experiments vertically in between two crossed polarizers. A Stocker & Yale Imagelite model 20 light source, color temperature 3200 K, was used, and the pictures were taken using a Canon EOS 550D camera with objective lens EFS 18–55 mm. Magnifying lenses with a 180-mm focus were also used in between the crossed polarizers.

### Atomic force microscopy

The surface topography has been determined by atomic force microscopic measurements. The images were acquired with a Dimension Icon (Bruker Nano Inc.) in PeakForce tapping mode in air. ScanAsyst Air cantilever (Bruker Nano Inc.) with a typical spring constant of 0.4 N/m and a resonant frequency of 70 kHz has been used. The PeakForce amplitude has been 60 nm and the PeakForce frequency has been 2 kHz. The AFM images were processed with NanoScope Analysis 1.80 (Bruker Nano Inc.). The topography was flattened by subtracting a first-order polynomial background using a threshold to exclude platelets from flattening. Platelet heights were determined by means of “step tool” in NanoScope Analysis software. The samples were prepared by slow evaporation of a few drops of a diluted suspension (0.02 g/liter) on a Si wafer under ambient conditions.

### Powder x-ray diffraction

PXRD (powder x-ray diffraction) of ordered interstratification was measured with textured samples in Bragg-Brentano geometry on a PANalytical X’pert Pro equipped with an X’Celerator Scientific Real Time Multiple Strip (RTMS) detector (CuKα radiation). The samples were equilibrated at least 24 hours at 43% relative humidity (RH) to build up the 1water layers (WL) around the Na^+^ cation.
