# Supplementary material for: Bright, noniridescent structural coloration from clay mineral nanosheet suspensions
Source: Sci Adv. 2022 Jan 26;8(4):eabl8147. doi: 10.1126/sciadv.abl8147 (PMC8791460; doi:10.1126/sciadv.abl8147)
Supplement: Supplementary file 1 — Supplementary Text Figs. S1 to S12 Legends for movies S1 to S3 [file sciadv.abl8147_sm.pdf]

Science Advances
AAAS

Supplementary Materials for

Bright, noniridescent structural coloration from clay mineral nanosheet suspensions

Paulo H. Michels-Brito, Volodymyr Dudko, Daniel Wagner, Paul Markus, Georg Papastavrou, Leander Michels, Josef Breu*, Jon Otto Fossum*

*Corresponding author. Email: josef.breu@uni-bayreuth.de (J.B.); jon.fossum@ntnu.no (J.O.F.)

Published 26 January 2022, *Sci. Adv.* **8**, eabl8147 (2022)
DOI: 10.1126/sciadv.abl8147

The PDF file includes:

Supplementary Text
Figs. S1 to S12
Legends for movies S1 to S3

Other Supplementary Material for this manuscript includes the following:

Movies S1 to S3

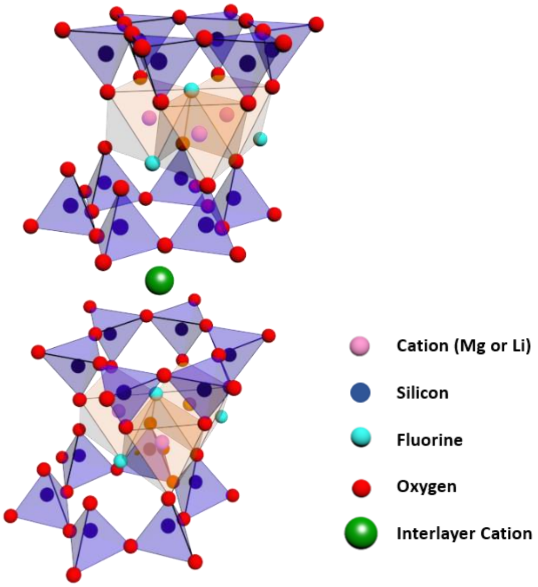

Fig. S1: **Na-Fluorohectorite structure.** The orange octahedral sites (pink sphere) contain magnesium partially substituted by lithium. The octahedral sheet is sandwiched in between the blue tetrahedral sheets. The tetrahedral sites (dark blue spheres) contain silicon. The light blue spheres are fluorine, and the red spheres are oxygen. The green spheres are the interlayer cations, typically Na^+ from the synthesis.

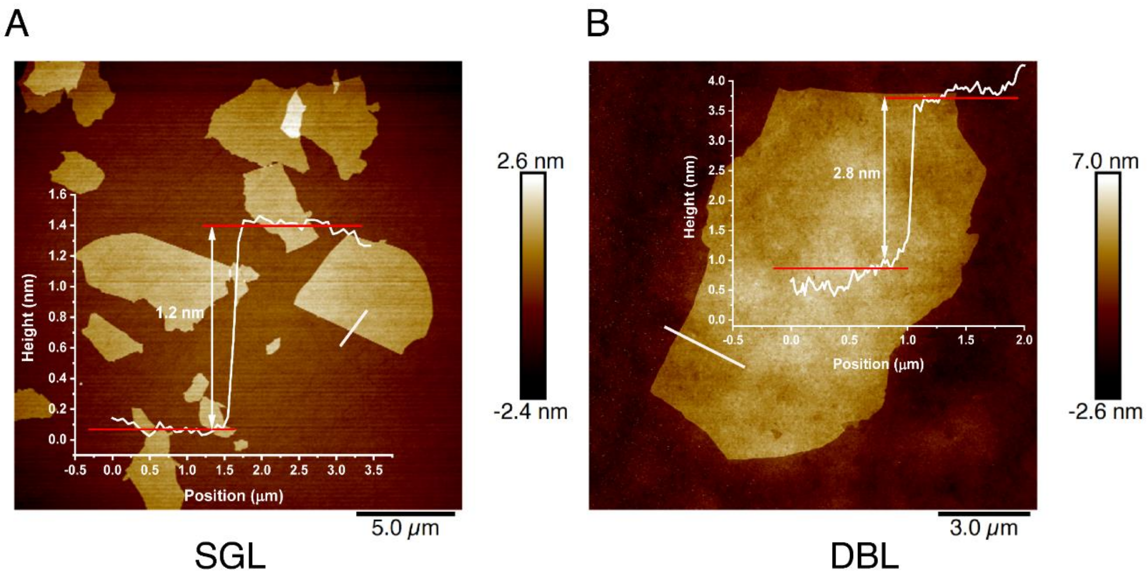

Figure. S2. Topographic AFM image of the SGL (A) and DBL (B) nanosheets. The height profiles show the height of 1.2nm for the SGL and 2.8 nm for the DBL.

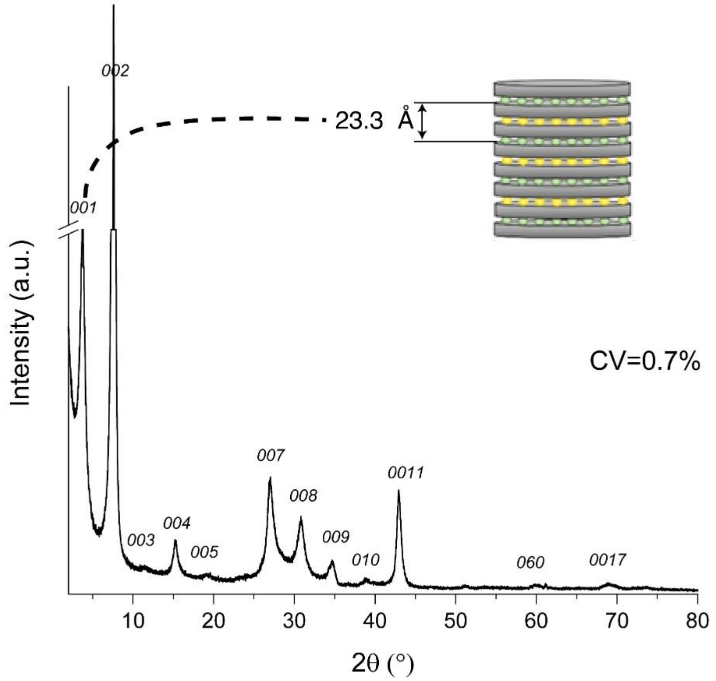

Figure S3. The PXRD pattern of the ordered interstratification of Na^+ and Cs^+ interlayers. The low (0.7%) coefficient of variation indicating the high degree of order in the alternating structure. The 001 peak is located in 2.3 nm, been a sum of Na^+ cation with interlayer water (1.2 nm) and Cs^+ interlayer which is not solvated with water (1.1 nm) see insert.

Fig. S4 displays the principle of reflective structural coloration obtained from a lamellar Bragg-stack suspension corresponding to a colloidal platelet smectic liquid crystalline phase, where the lamellar distances match the wavelength of the incoming white light. The interference of the reflected white light from all layers and the effect of the refractive index included in the Bragg-Snell's equation $2d(n^2 - sin^2\theta)^{1/2} = m\lambda$, where d is the lamellar distance, θ is the angle between the observer's point of view and the lamella horizontal, n is the effective refractive index, in the present case a weighted combination of the water the refractive index and the clay lamella refractive index, m is the order of the Bragg-Snell reflection and λ is the wavelength of the light enhanced by constructive interference for a given d and θ. The effect of the refractive indices modifies the light paths according to the Bragg-Snell´s law.

A) **B)**

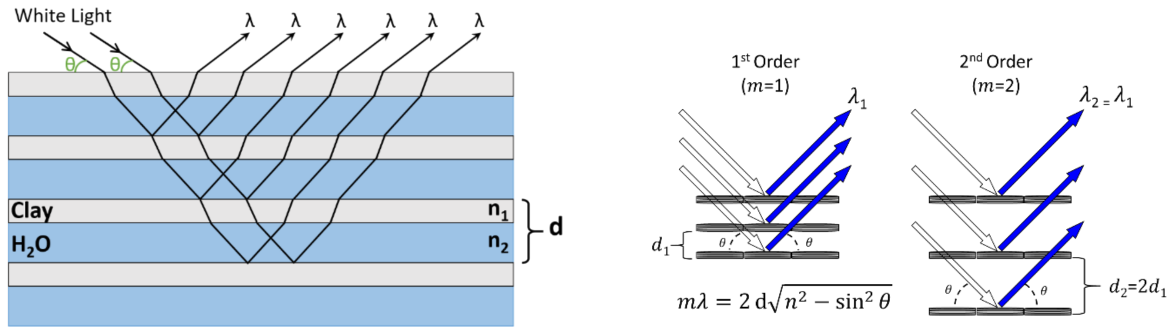

Figure S4: Principle of reflective structural coloration obtained from a lamellar Bragg-stack suspension. A) How the refractive index modifies the light paths described by the Bragg-Snell Law, where the effective refractive index n is a weighted combination of n_1 and n_2. **B)** Illustration of the effect of the order factor (m).

Small Angle X-ray Scattering (SAXS) of DBL suspensions were recorded for a series of suspensions with d-spacings giving peaks inside the instruments accessible q-range., see Fig. S5. We observed a series of *00l*-reflections indicating the formation of excellent 1D crystalline order of Bragg stacks in the DBL suspension. With increasing concentration, the peak positions shift to high q-values, indicating a reduction of separation between the adjacent DBL nanosheets. The first minimum of the form factor oscillation was observed at 2.1 ± 0.1 nm corresponding to sum of 2 SGL of 0.85 nm and an interlayer height of 0.4. Independent of clay concentration, all scattering curves showed this same minimum, see Fig. S5A, which confirms that all nematic suspensions contain same DBL nanosheets separated to different distances. In principle, the effective refractive index could be determined using SAXS and RSP data in the Bragg-Snell law, but the broad SAXS peaks (Fig. S5B) with corresponding large uncertainties in the estimate of d makes this impossible. Fig. S5C is merging of Fig S5A and B for the most concentrated sample (7.21%).

A)

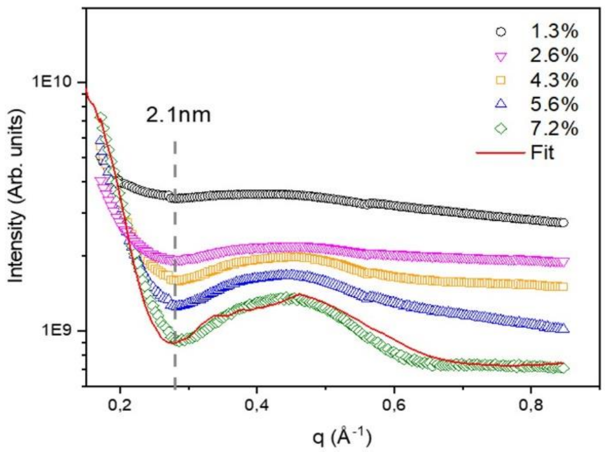

B)

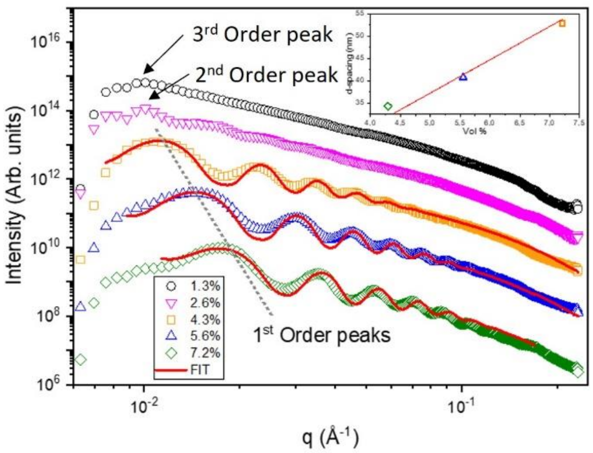

C)

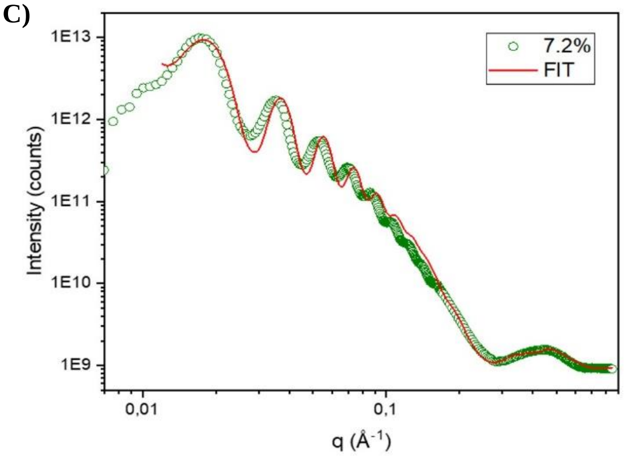

Figure S5: SAXS data of DBL suspensions for different volume percentages Φ. A) SAXS data with detector positioned at 0.2 meters. The minima marked by grey dash line represent the DBL thickness of 2.1 ± 0.1 nm obtained from fit of most concentrated sample (7.2%) using SasView model cylinder of stacked (bilayer) disks. **B)** SAXS data with detector positioned at 1 meter. The samples 4.3, 5.6 and 7.2% were fitted using SasView model lamellae of lamellar stack paracrystal. The inset displays the resulting d-spacing vs. Vol%. **C)** Merging A and B for the highest concentrated sample (7.2%).

Birefringence (BF) measurements were performed in sequence immediately after RSP characterization. In Fig. S6 we show the full range of structural color and corresponding BF for the first and second order colors. All BF images show a vertical narrow feature that comes from the syringe needle sample insertion. The first order presented more uniform colors of DBL in suspension than second order color. With decreasing concentration, the second-order colors, DBLs have more translational and rotational freedom giving less orientational order and producing asymmetrical structural colors. This reduction could render non-uniform distribution of DBLs in suspension yielding regions with different d-spacings and thus heterogeneous structural colors. This is also evident for the RSPs in the R2 range, where the spectra for each sample have broader spectral maxima, and in some cases more than one peak in the same sample.

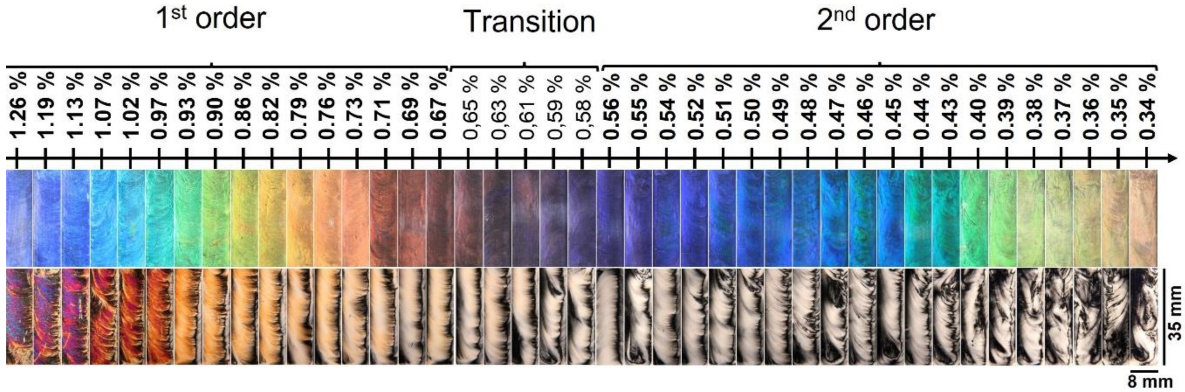

Figure S6: Full range and spectra transition, and the birefringence (BF). The displayed cuvettes are about 1cm x 6cm.

The spectral maxima positions were determined by fitting a second-degree polynomial both for first and second order colors. First, the spectra are normalized, and then the peak wavelength was chosen at the polynomial spectral maxima position, see Fig. S7.

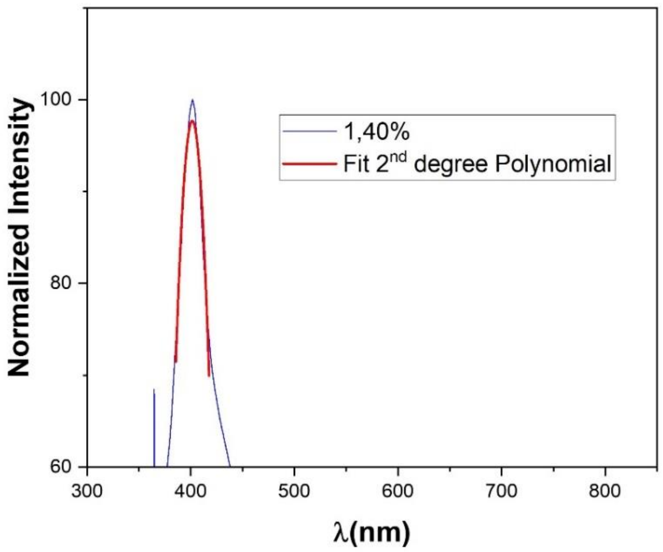

Figure S7: Spectrum of 1.40 vol % sample. The fit was done using 2nd degree polynomial.

Samples began to exhibit some iridescence s after some resting time. This is shown in Fig. S8 below.

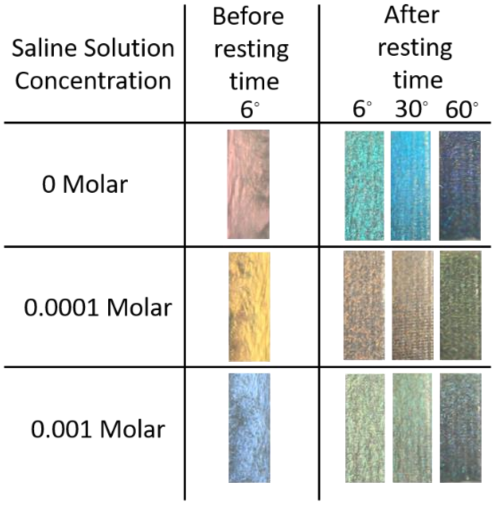

Figure S8: The samples exhibit iridescence after some resting time. The sample in deionized water (0 Molar) started to exhibit some iridescence after resting for 4-5 days and samples in saline solution after resting 48 hours. DBL concentration 0.68% was used for all displayed samples. The cuvettes are about 1cm x 6cm, and with 1 mm pathlength.

Colors were also obtained in 200 µm pathlength cuvettes, see Fig. S9 below.

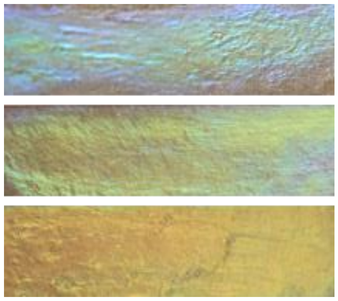

Figure S9: Preliminary results on colors obtained in 200 µm pathlength cuvettes. Based on the same stock suspension used for the 1 mm pathlength (1 cm x 6 cm) cuvettes.

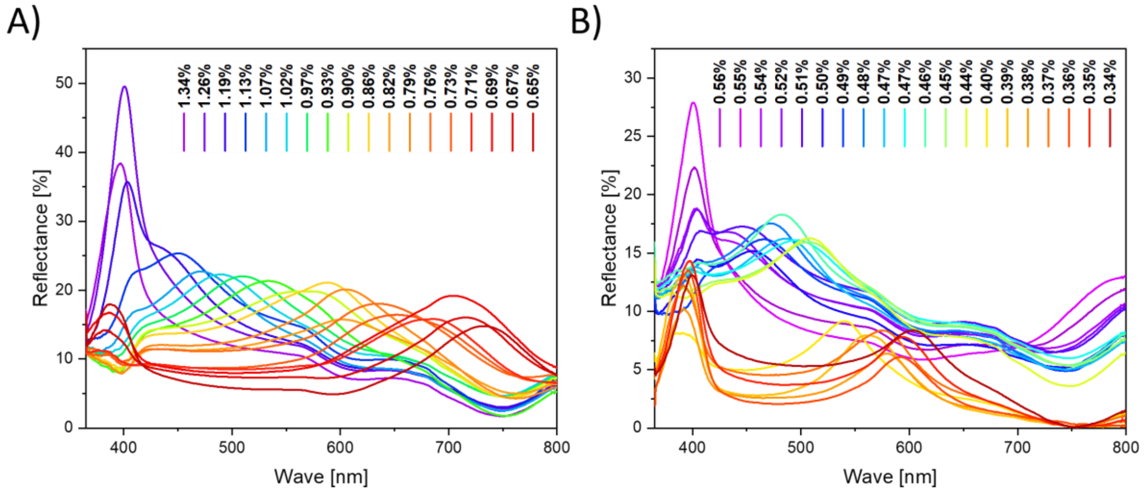

Figure S10: **Normalization based on the black and white background references.** For clarity, normalization with respect with maximum intensity for each color is displayed in Fig. 2B and Fig. 2C.

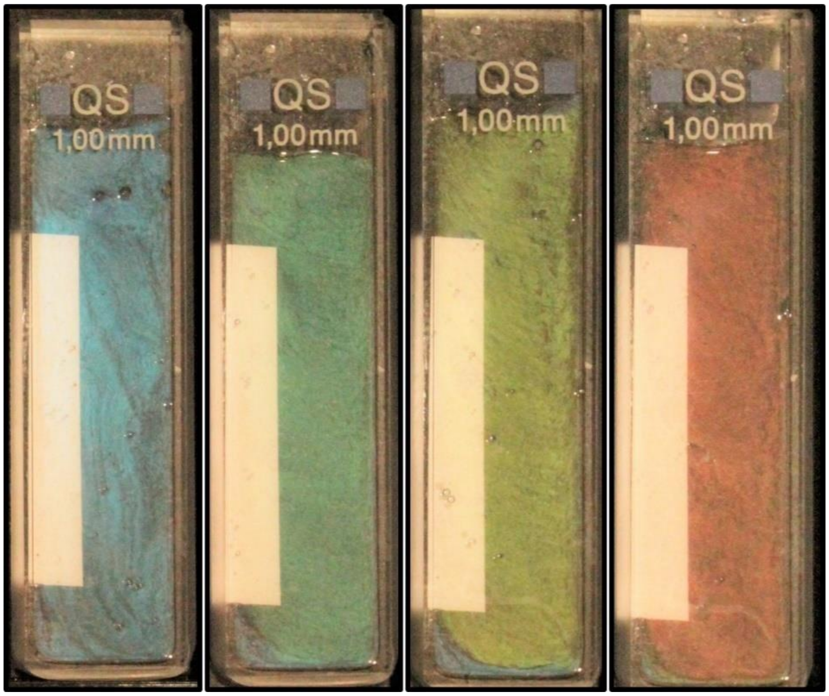

Figure S11: The transparency of structural colors. The colors are placed on a black background with a rectangular white background partly inserted from the side.

A)

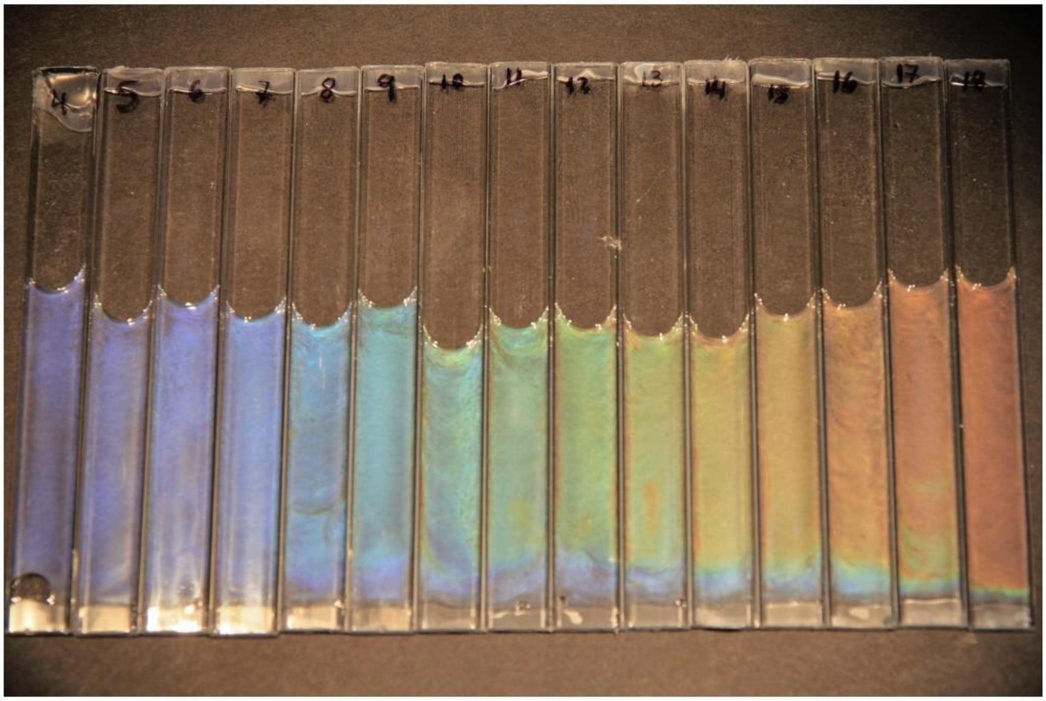

B)

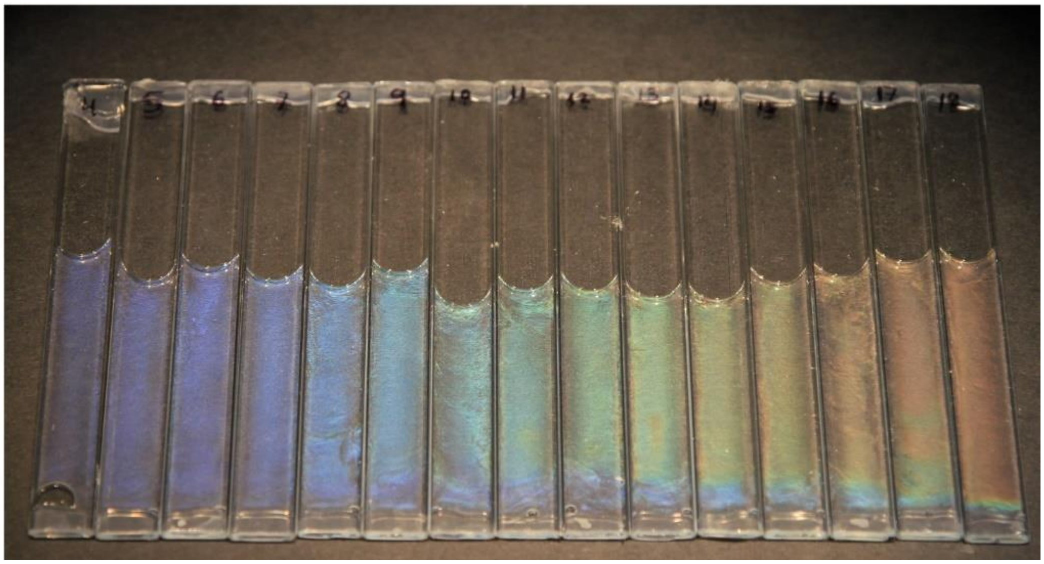

Figure S12: **Non-iridescence of the first order structural colors. A)** Structural colors at angle of 5° approximately. **B)** Same structural colors at angle of 30° approximately.

Video S1.

Visualization of three examples structural colors in quartz cuvettes on top of a dark background with translational movement.

Video S2.

Visualization of the change from the black background to white background under the structural colors in quartz cuvette.

Video S3.

Tunability of strutural color by adding water in the suspenstion. In half of the quartz cuvette is inserted a clay double layer suspension with a light blue color. The other half is filled with water. After mixed with the syringe needle, the sample presented a wide range of structural color. This diversity of structural colors are a result of the incomplete homogenization of the suspension, which resulted in regions with different concentrations across the sample inside the cuvette resulting in a broad range of structural colors.
